# Anethole prevents hydrogen peroxide-induced apoptosis and collagen metabolism alterations in human skin fibroblasts

**DOI:** 10.1007/s11010-014-2097-0

**Published:** 2014-06-05

**Authors:** Anna Galicka, Rafał Krętowski, Jolanta Nazaruk, Marzanna Cechowska-Pasko

**Affiliations:** 1 Department of Medical Chemistry, Medical University of Bialystok, Białystok, Poland; 2 Department of Pharmaceutical Biochemistry, Medical University of Bialystok, Białystok, Poland; 3 Department of Pharmacognosy, Medical University of Bialystok, Białystok, Poland

**Keywords:** (*E*)-Anethole, Hydrogen peroxide, Collagen, Matrix metalloproteinases, Apoptosis, Human skin fibroblasts

## Abstract

The collagen metabolism alterations triggered by reactive oxygen species are involved in the development of various connective tissue diseases and skin aging. This study was designed to examine whether (*E*)-anethole possesses a protective effect on H_2_O_2_-induced alterations in collagen metabolism as well as whether it can prevent apoptosis in human skin fibroblasts. In cells treated with 300 µM H_2_O_2_, a decrease in collagen biosynthesis of 54 % was observed. Pretreatment of cells with 0.5 µM anethole for 1 h completely prevented this alteration. Changes at the protein level positively correlated with alterations of type I collagen mRNA expression. We have shown that H_2_O_2_ caused increase in the activity of MMP-2 and MMP-9 as well as that an increase in MMP-2 activity can contribute to the 8 % decrease in the amount of collagen secreted into the medium. The most efficient suppression of these changes was observed in the presence of 0.5 µM of anethole. At 10 µM, in addition to suppression, an inhibitory effect of anethole on MMP-9 activity was documented. Additionally, the 60 % H_2_O_2_-induced decrease in cell viability was suppressed by 1 µM of anethole and a 4-fold increase in cell apoptosis was suppressed by 0.5 µM of anethole. Our results suggest that anethole, which is a small lipophilic and non-toxic molecule with the ability to prevent H_2_O_2_-induced collagen metabolism alterations and apoptosis in human skin fibroblasts, would prove useful in the development of effective agents in pharmacotherapy of oxidative stress-related skin diseases.

## Introduction

Hydrogen peroxide, like other reactive oxygen species (ROS), is generated at low levels during normal cellular metabolism. However, there is a lot of evidence for its damaging effects at higher concentrations on cells and components of the extracellular matrix (ECM) [1]. Because of its small size, its solubility and its lack of charge, H_2_O_2_ easily penetrates into cells and interacts with intracellular ions such as iron and copper, generating highly reactive radicals. These induce damage of cellular biomolecules such as lipids, nucleic acids, and proteins and are implicated in the pathogenesis of various human degenerative connective tissue diseases and pathological processes, including carcinogenesis or human skin aging [2, 3]. Alterations in the synthesis and degradation of collagen, the main component of extracellular matrix, triggered by ROS, may be responsible for the development of these pathological changes. Therefore, agents with the ability to scavenge ROS might be beneficial in the treatment of different diseases as well as in protecting health.

The most beneficial effects against diseases such as cancer, cardiovascular diseases, and neurodegenerative disorders were shown by dietary polyphenols, which have been widely studied for their strong antioxidant properties [1, 4]. Essential oil constituents, especially phenolic volatile compounds also show antioxidant activity. They work due to their high reactivity with peroxyl radicals [5].

**Fig. 1:**
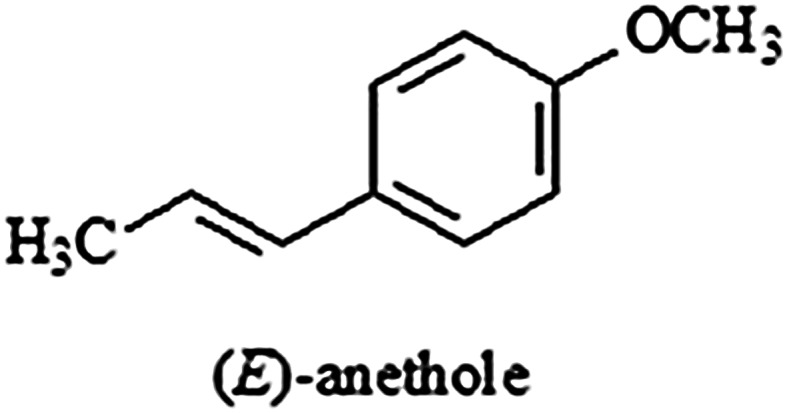
Chemical structure of anethole

Essential oils are still a poorly recognized group of natural compounds with great biologic potential. Interesting properties have been demonstrated by (*E*)-anethole (later called anethole) (Fig. 1). This phenylpropanoid derivative [1-methoxy-4-(1-propenyl)benzene] is a major component of anise and fennel fruit essential oils, where its content is higher than 80 %. These essential oils are traditionally used in herbal medicine as expectorant and carminative drugs [6]. Contemporary studies show that anethole inhibits TNF-induced cellular responses, which leads, among others, to the suppression of inflammation and carcinogenesis. This compound also inhibits the production, or release, of cytokines, prostaglandins, and nitric oxide [7, 8].

It has been reported that, among its multidirectional action, anethole also exhibited antioxidant properties and caused moderate suppression of lipid peroxidation [9]. Its antioxidant activity, similar to other phenolic compounds, depends on the conjugate double bonds [7]. The aim of this study was to examine whether anethole has a protective effect on H_2_O_2_-induced alterations in collagen metabolism, as well as if it can prevent the apoptosis of human skin fibroblasts.

## Materials and methods

### Plant material

Anethole used for the study was obtained from a commercial anise fruit essential oil (Pollena-Aroma, Warsaw, Poland) using flash chromatographic separation on a silica gel column eluted with hexane and mixtures of hexane and diethyl ether (increasing polarity). Separation was monitored using gas chromatography (GC) and gas chromatography-mass spectrometry (GC–MS), and the purity of the obtained compound reached 99 %. GC and GC–MS were performed on a Perkin–Elmer AutoSystem XL equipped with a Perkin–Elmer TurboMass detector and a Perkin–Elmer Elite 5MS column, 30 m × 250 µm I.D., 1 µm film thickness. Identification was carried out on the basis of comparing the mass spectrum of the compound with the mass spectrum listed by the NIST MS Library.

### Fibroblast cultures

Human normal skin fibroblast cell line (CRL1474) was obtained from the American Type Culture Collection (ATCC). Cells were maintained in Dulbecco’s modified Eagle’s medium (DMEM) supplemented with 10 % heat-inactivated fetal bovine serum GOLD (FBS GOLD), 2 mM glutamine, penicillin (100 U/ml), and streptomycin (100 μg/ml). Cells were cultured in Falcon flasks (BD) in a 5 % CO2 incubator (Galaxy S+), at 37 °C. Subconfluent cultures were detached with 0.05 % trypsin, 0.02 % EDTA in calcium-free phosphate-buffered saline and counted in a Scepter cell counter (Millipore).

### Estimation of biologic action of anethole in H_2_O_2_-treated and untreated skin fibroblasts

Cells (2.5 × 10^5^) were seeded in six-well plates. Confluent cells were preincubated in a fresh serum-free medium for 2 h. The anethole dissolved in DMSO was added to the medium to a final concentration of 0.5, 1, and 10 μM and incubated for 24 h. The same concentration of DMSO solution (0.01 %) was used as control in order to rule out the possible effect of DMSO on fibroblasts. In experiments on the protective role of anethole against the destructive action of H_2_O_2_, cells were treated with 300 µM of H_2_O_2_ for 24 h or, prior to H_2_O_2_ delivery, pretreated with anethole for 1 h and incubated over 24 h. After incubation, the exposure medium was removed and stored for analysis of collagen content and matrix metalloproteinases (MMPs) activity. The monolayers were washed three times with sterile 10 mM PBS pH 7.4, and cell membranes were disrupted using sonicator (Sonics Vibra cell). Aliquots of the homogenate were used for collagen and protein measurement as well as for RNA isolation. A BCA Protein Assay Kit (Pierce) was used for a protein concentration measurement.

### Collagen biosynthesis assay

5 μCi L-[5-^3^H]Proline (28 Ci/mmol) was added to skin fibroblast cultures treated with only H_2_O_2_ and to one pretreated with anethole, and then incubated for 24 h. Incorporation of radioactive precursor into collagen was determined by digestion of the proteins with purified *Clostridium histolyticum* collagenase in accordance with the method developed by Peterkofsky et al. [10]. Secretion of collagen was estimated as the distribution of protein between the cell layer and the medium.

### MMPs assay

Gelatinolytic activity of the media was determined according to the method of Unemori and Werb [11]. Equal amounts (10 μg) of protein were electrophoresed under non-reducing conditions in 10 % polyacrylamide gel impregnated with 1 mg/ml gelatin (Sigma) as a substrate. After electrophoresis the gel was washed twice for 15 min with 2 % Triton X-100 and then incubated overnight at 37° C in 50 mM Tris/HCl, pH 8.0, containing 5 mM CaCl_2_. The gel was stained with 0.5 % coomassie brilliant blue R-250. Clear bands on the blue background, representing areas of substrate-degrading enzymes, were quantified using an imaging densitometer (G:BOX, Syngene).

### Real-time PCR

Total RNA was isolated using the MasterPure^TM^ RNA purification kit (Akor Laboratories). The RNA extracts were treated with RNase-free DNase Ι to remove contaminating DNA, quantified on a spectrophotometer (Nanodrop 2000, ThermoScientific) and stored at –80 °C. Real-time PCR assays performed in CFX96 Real-time system (Bio-Rad) were used to quantify mRNA levels of type I collagen. The gene GAPDH (glyceraldehyde-3-phosphate dehydrogenase) was evaluated as housekeeping. Total RNA (1 µg) in the total volume of 20 μl was reverse transcribed using a Tetro cDNA Synthesis Kit (Bioline) and 1 µl oligo(dT) primer. Real-time PCR was carried out using 2 µl of the cDNA product, 400 nM each of the primer and the SensiFAST™ SYBR Kit (Bioline). The primers used for type I collagen (*COL1A1* gene) were: forward 5′-ATG TCT AGG GTC TAG ACA TGT TCA-3′, reverse 5′-CCT TGC CGT TGT CGC AGA CG-3′ and for *GAPDH* they were: forward 5′-CAT GAC AAC TTT GGT ATC GTG G-3′ and reverse 5′-CCT GCT TCA CCA CCT TCT TG-3′ [12]. Cycling parameters were: 95 °C for 1 min to activate the DNA polymerase, then 40 cycles of denaturation for 10 s at 95 °C, annealing for 15 s at 60 °C, and extension for 20 s at 72 °C. The reaction was then subjected to a melting protocol from 55 °C to 95 °C with a 0.2 °C increment and 1 s holding at each increment to check the specificity of the amplified products. Single product formation was confirmed by melting point analysis and agarose gel electrophoresis. For negative control, water instead of mRNA samples was used. Samples were run in triplicate and the ΔΔCT method was applied for statistical analysis of the CT-values. The relative gene expression levels were standardized with those measured in the untreated control.

### Assay for cell viability

The assay was performed according to the method developed by Carmichael et al. [13] using MTT [3-(4,5-dimethylthiazol-2-yl)-2,5-diphenyltetrazolium bromide]. Briefly, cells were seeded in a 24-well plate at a density of 10^4^ per well. Confluent cells cultured with tested compounds for 24 h at 37 °C were washed three times with PBS and then incubated for 4 h with 1 ml of MTT solution (0.25 mg/ml in PBS). The medium was removed, and 1 ml of 0.1 M HCl in absolute isopropanol was added. Absorbance of converted dye in living cells was measured at a wavelength of 570 nm.

### Detection of apoptosis

Apoptosis was evaluated using flow cytometry on a FACSCanto II cytometer (Becton–Dickinson). Cells were trypsinized, resuspended in DMEM and then in a binding buffer. Next, the cells were stained with FITC Annexin V and PI for 15 min at room temperature in the dark following the manufacturer’s instructions (FITC Annnexin V apoptosis detection Kit I). Data were analyzed with FACSDiva software and dead cells were excluded based on forward- and side-scatter parameters.

### Statistical analysis

In all the experiments, the mean values for three assays ± SD were calculated. The results were subjected to statistical analysis using the one-way analysis of variance (ANOVA) followed by the Duncan’s multiple range post hock test. Differences were recognized as statistically significant at *P* < 0.05. Spearman rank correlation analysis was conducted to investigate the relationship between the degrading of collagen enzymes and collagen expression in the media. Correlations were considered statistically significant at *P* < 0.05. All the calculations were performed using the Statistica 9.0 package (StatSoft, Tulsa, OK, USA).

## Results

### The effects of H_2_O_2_ treatment on cell viability

**Fig. 2:**
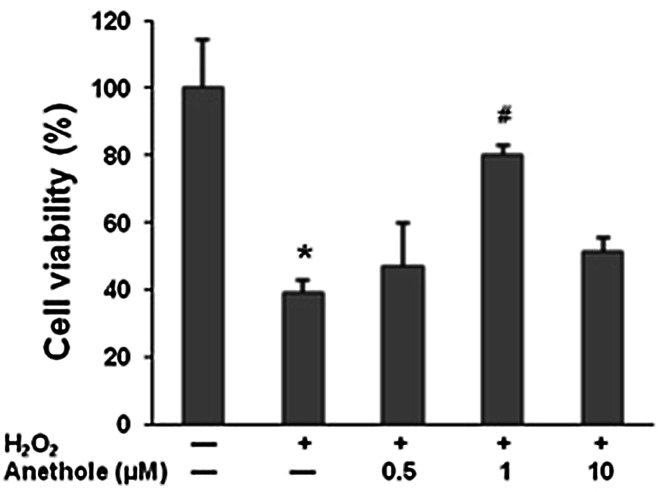
Effect of anethole on H_2_O_2_-induced cytotoxicity in human skin fibroblasts. Cells were pretreated with anethole for 1 h and then exposed to 300 µM H_2_O_2_ for 24 h. Values ± standard deviation (SD) are the mean of triplicate cultures.*P < 0.01, no treatment versus control H_2_O_2_; ^#^ *P* < 0.01, control versus anethole

Treatment of human skin fibroblasts with 300 µM of H_2_O_2_ induced a decrease of cell viability by 60 % as compared to the control (Fig. 2). Pretreatment of cells prior to H_2_O_2_ delivery with 1 µM of anethole caused a 2-fold increase in cell growth compared to H_2_O_2_ treatment alone, suggesting that anethole suppressed the H_2_O_2_-induced cytotoxicity. Anethole used at a higher concentration of 10 μM prevented cytotoxicity to comparable extent as 0.5 μM of the compound. Furthermore, anethole alone did not affect viability of cells at concentrations up to 100 μM (data not shown).

### Suppression of H_2_O_2_-induced decrease in collagen biosynthesis and MMPs activity increase

**Fig. 3:**
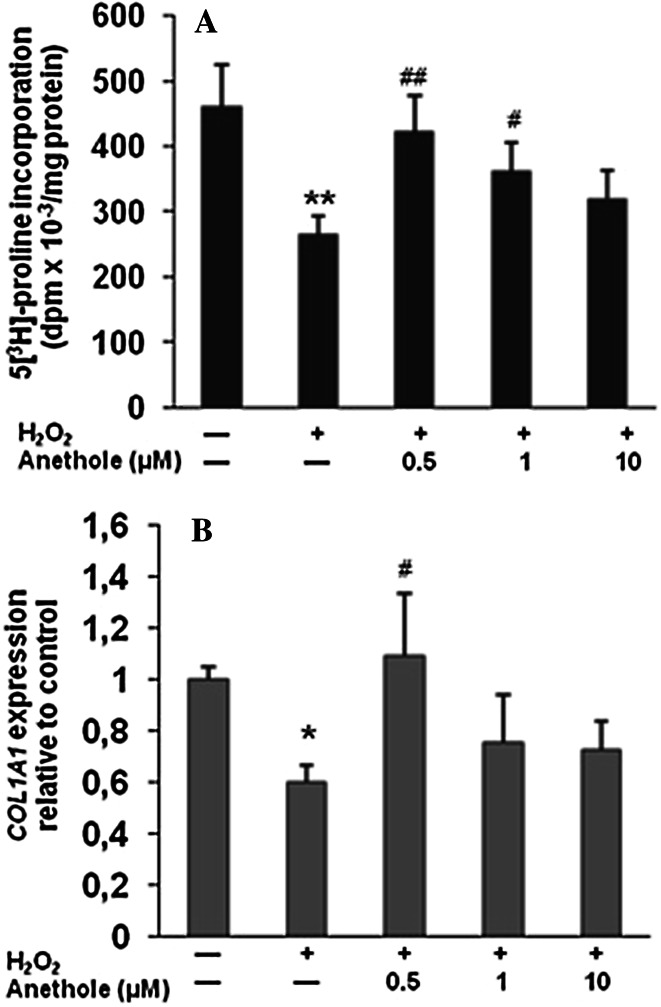
Effect of anethole on H_2_O_2_-induced changes in collagen biosynthesis (**A**) and the expression of *COL1A1* gene (**B**) in human skin fibroblasts. Cells were incubated with anethole for 1 h and then exposed to 300 µM H_2_O_2_ for 24 h. *P < 0.05, **P < 0.01 no treatment versus control H_2_O_2_; ^#^ *P* < 0.05, ^##^ *P* < 0.01 control versus anethole. Values ± standard deviation (SD) are the mean of triplicate cultures

**Fig. 4:**
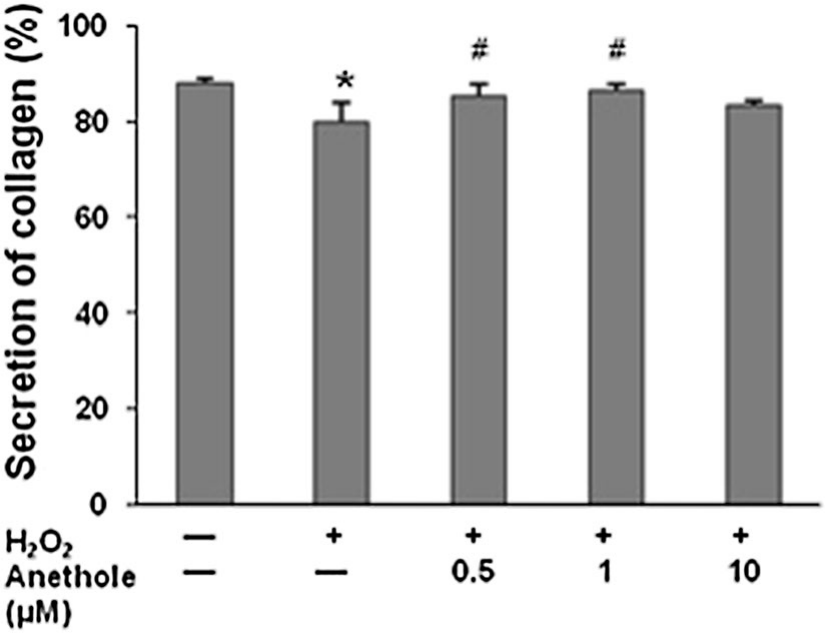
Effect of anethole on collagen secreted into media in the presence of H_2_O_2_. Skin fibroblasts were pretreated with anethole for 1 h and then exposed to 300 µM H_2_O_2_ for 24 h. Values ± standard deviation (SD) are the mean of triplicate cultures. *P < 0.01, no treatment versus control H_2_O_2_; ^#^ *P* < 0.05, control versus anethole

Collagen biosynthesis in skin fibroblasts was measured using L-[5-^3^H]proline incorporation into proteins [10]. In cells treated with 300 µM of H_2_O_2_, a significant decrease in collagen biosynthesis (by 54 %) as compared to untreated cells was demonstrated (Fig. 3A). Pretreatment of cells prior to H_2_O_2_ delivery with anethole prevented collagen biosynthesis decrease. With anethole at concentrations of 0.5 and 1 µM, a significant increase in collagen content, by 59.6 and 36.5 %, respectively, compared to H_2_O_2_ treatment alone, was observed. The results of the protective effect of anethole on collagen in H_2_O_2_-treated cells at the protein level positively correlated with its effect on collagen at the mRNA level (*r* = 0.528, *P* < 0.05), as was assessed using real-time PCR (Fig. 3B). Secretion of collagen was estimated as the distribution of protein between the cell layer and the medium. The percentage of collagen secreted into the medium was estimated at 87 % for the untreated cells. We observed a decrease in collagen secretion of up to 79 % for cells treated with 300 µM of H_2_O_2_ (Fig. 4). Exposure of the cells to 0.5 and 1 µM of anethole before treatment with H_2_O_2_ resulted in the normalization of the amount of collagen secreted into the medium.

**Fig. 5:**
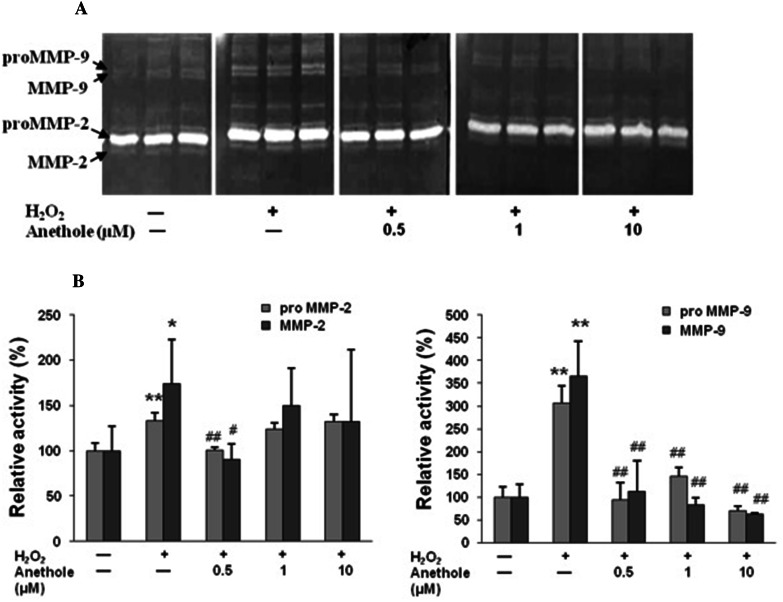
Protective effect of anethole on H_2_O_2_-induced increase in gelatinases activity in human skin fibroblasts. **A** Representative zymography of pro- and active forms of MMP-2 and MMP-9. **B** Densitometric intensity of the zymography bands expressed as percentage of the control. Skin fibroblasts were pretreated with anethole for 1 h and then exposed to 300 µM H_2_O_2_ for 24 h. Values ± standard deviation (SD) are the mean of triplicate cultures. *P < 0.05, **P < 0.01 no treatment versus control H_2_O_2_; ^#^ *P* < 0.05, ^##^ *P* < 0.01 control versus anethole

It has been shown that ROS cause an increase in the activity of matrix metalloproteinases (MMPs) which degrade collagen in skin fibroblasts [14, 15]. Therefore, we treated cells with 0.3 mM H_2_O_2_ and studied whether anethole can protect them against changes in MMPs activity induced by H_2_O_2_. Using zymography and gelatin as a substrate, we detected the presence of pro-MMP-2 (72 kDa) and pro-MMP-9 (95 kDa) and their active forms (66 kDa) and (88 kDa), respectively (Fig. 5A). As was demonstrated by densitometry, H_2_O_2_ caused an increase in the activity of both forms of MMP-2 (72 and 66 kDa) by 33 and 73 %, respectively (Fig. 5B). After pretreatment of cells with anethole, the greatest protection of pro-MMP-2 and its active form against H_2_O_2_ action was demonstrated at concentration of 0.5 μM (a decrease of 33 and 83 %, respectively). Negative correlations occurred between collagen measured using L-[5-^3^H]proline incorporation (Fig. 3A) and pro-MMP-2 (*r* = −0.828, *P* < 0.01), as well as its active form (*r* = −0.639, *P* < 0.05) (Fig. 5).

Similarly to MMP-2, we observed an influence of H_2_O_2_ on both forms of MMP-9 (95 and 88 kDa), however in a much more drastic way (a 3- and 3.6-fold increase, respectively) (Fig. 5B). Anethole used at all concentrations significantly inhibited the increase in the intensity of both bonds corresponding to the molecular masses of both forms of MMP-9. However, the most efficient suppression was observed at 0.5 µM. It is worth adding that anethole at concentration of 10 µM inhibited activity of 95 and 88 kDa of MMP-9 by 30 and 38 %, respectively, in relation to untreated controls. In contrast to MMP-2, no significant correlations between synthesized collagen and MMP-9 activity were detected.

### The effect of anethole on H_2_O_2_-induced apoptosis

**Fig. 6:**
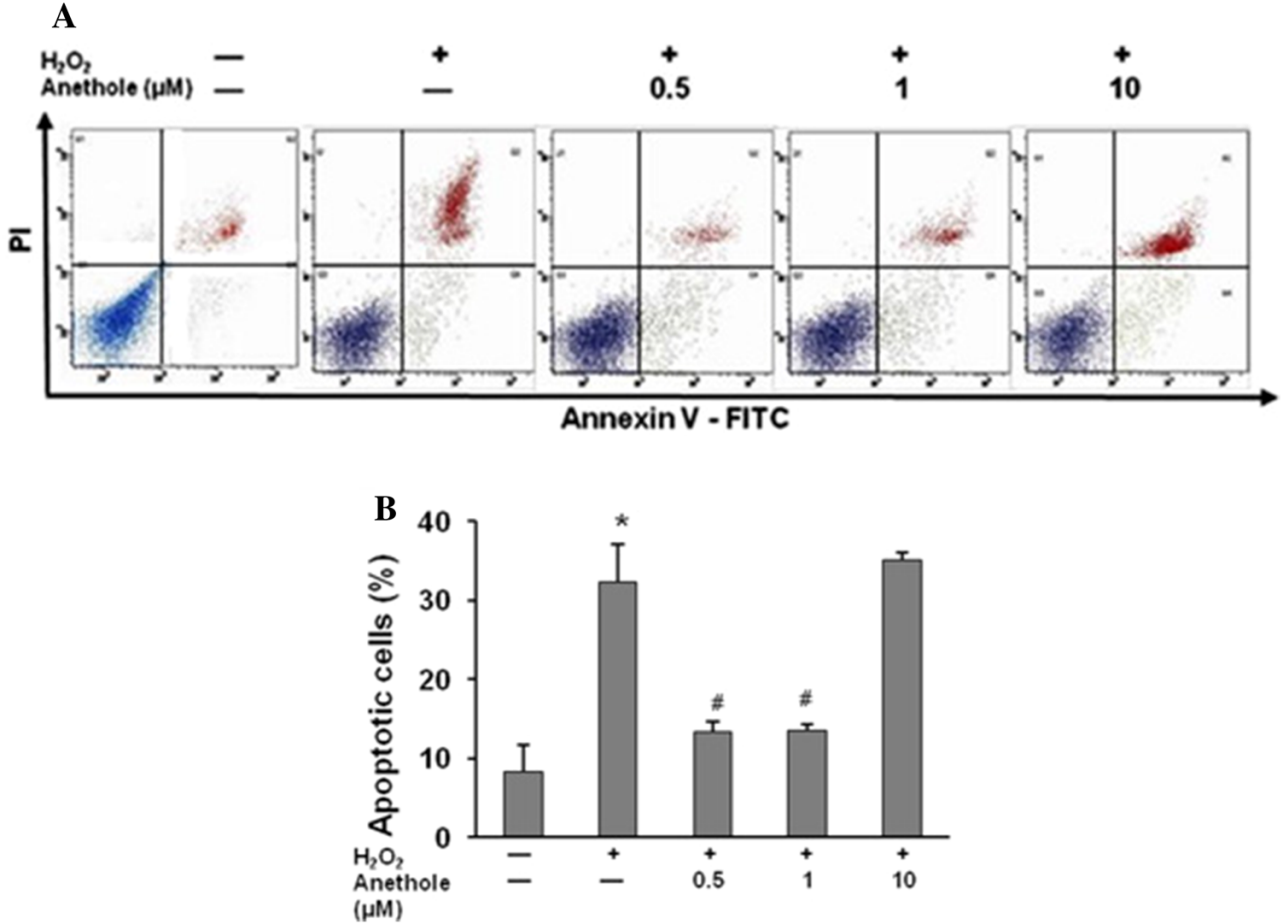
Effect of anethole on H_2_O_2_-induced apoptosis of human skin fibroblasts. **A** Representative flow cytometric analysis using two color staining with annexin V-FITC and PI. **B** Percentage of apoptotic cells. Cells were pretreated with anethole for 1 h and then exposed to 300 µM H_2_O_2_ for 24 h. Values ± standard deviation (SD) are the mean of triplicate cultures. *P < 0.01 no treatment versus control H_2_O_2_; ^#^ *P* < 0.01 control versus anethole

It has been reported that hydrogen peroxide, like other ROS, can increase the apoptosis of human skin fibroblasts [16, 17]. Apoptosis was estimated in cells treated with H_2_O_2_ and pretreated with anethole at different concentrations prior to H_2_O_2_ delivery using an FITC Annexin V and flow cytometry detection. Incubation of fibroblasts with 300 µM of H_2_O_2_ for 24 h resulted in a 4-fold increase in the percentage of apoptotic cells (Fig. 6). It is worthy of note that anethole, at concentrations of 0.5 and 1 µM but not at 10 µM, significantly decreased apoptosis in comparison to cells incubated with H_2_O_2_.

## Discussion

Oxidative stress can be generated in the connective tissue of the skin during its UV radiation, inflammatory processes like wound healing, and in skin aging [1–3]. The most promising treatments of these pathological changes include herbal extracts, vitamins, and antioxidant food supplements, which have been reported widely to scavenge free radicals from skin cells [1, 4, 17, 18].

It has been shown that hydrogen peroxide, like other reactive oxygen species, plays a substantial role in the metabolism of the main component of ECM, collagen [14, 19, 20]. We also found that hydrogen peroxide used at concentration of 0.3 mM caused a decrease in collagen biosynthesis in human skin fibroblasts by 54 %. Pretreatment of cells with anethole at a low concentration of 0.5 µM completely prevented this alteration. These changes at the protein level were correlated with alterations in the mRNA expression of type I collagen, and is in agreement with other studies [14, 20]. Anethole at the concentration of 0.5 µM totally abrogated the H_2_O_2_-induced alteration of *COL1A1* gene.

Extracellular collagen plays an important role in the maintenance of the structural integrity of ECM, and its level is determined by the balance between synthesis and degradation [21]. MMPs, which are zinc-dependent endopeptidases, degrade components of ECM and, therefore, play an important role in physiologic and pathological remodeling [22]. MMP-2 (gelatinase A) and MMP-9 (gelatinase B) are key enzymes in the degradation of ECM collagen and are regulated through activation of latent proenzymes (pro-MMPs).

In our study we have shown that H_2_O_2_ exhibited a stimulating effect on the activity of both MMP-2 forms (72 and 66 kDa) (33 and 73 %, respectively) and that 0.5 µM of anethole completely protected against these changes. These results suggest that the effect of hydrogen peroxide was mediated by the induction of MMP-2 synthesis and activation at the translational and post-translational level. There is evidence that H_2_O_2_ is involved in the induction of MMP-2 at the mRNA level [14, 15]. Furthermore, the authors reported that H_2_O_2_ not only directly activates MMPs, but also causes a decrease in the expression of their inhibitors, such as TIMP2. The significant negative correlations between collagen content and MMP-2 activity, which have been found in our study, suggest that the increase in enzyme activity can contribute to the decrease in collagen synthesized in H_2_O_2_-treated cells. MMP-2 is known to digest native type I collagen and generate the 3/4- and 1/4-fragments characteristic of vertebrate collagenases [23].

Similarly, both forms of MMP-9 (95 and 88 kDa) were significantly influenced by hydrogen peroxide, much more than MMP-2, since a 3- and 3.6-fold increase in their activity, respectively, was observed. Anethole at all concentrations used (0,5, 1, and 10 µM) significantly inhibited the increase in the intensity of both bonds corresponding to the molecular masses of MMP-9, with the most efficient suppression demonstrated at 0.5 µM. Furthermore, anethole at concentration of 10 µM inhibited the activity of both 95 and 88 kDa MMP-9 by 30 and 38 %, respectively, in comparison to the untreated control. It has been reported that anethole used at a higher concentrations of 50 and 100 µM inhibited the activity of both MMP-2 and MMP-9 in HT 1080 cells suggesting its antimetastatic activity [24]. However, in contrast to MMP-2, no significant correlations between collagen content in the medium and MMP-9 activity were detected.

We also examined the effect of anethole on H_2_O_2_-induced cytotoxicity and apoptosis of skin fibroblasts. Treatment of cells with 0.3 mM H_2_O_2_ significantly decreased viability of cells compared with the control cultures, confirming previous data of its toxic effect on fibroblasts [15, 25, 26]. Anethole significantly suppresses the H_2_O_2_-induced cytotoxicity at a concentration of 1 μM. We did not observe a more efficient prevention at higher concentrations. Several authors have reported that hydrogen peroxide can induce apoptosis in fibroblast cell cultures [16, 17, 26]. In our study, after a 24 h treatment of cells with 300 µM of H_2_O_2_, the percent of apoptotic cells increased 4-times and apoptosis was significantly attenuated by anethole at concentrations of 0.5 and 1 µM.

It is well known that a decrease in the content of dermal collagen, a major ECM protein, results in the loss of tensile strength and elasticity of skin, increases its fragility, and impaired wound healing, all of which are characteristic of aged skin. Skin aging has general relevance for many degenerative connective tissue diseases such as osteoarthritis, osteoporosis, and arteriosclerosis [1–3]. Therefore, agents with the ability to scavenge ROS, elevate ECM collagen levels or inhibit major collagen-degrading enzymes, would be useful in the development of effective agents in pharmacotherapy of various connective tissue diseases.

Components of essential oils containing a phenol group in their structure have considerable antioxidant properties. It has been shown in experiments that the volatile fractions of *Thymus *sp. and *Eugenia* sp. and their main components thymol and eugenol are strong antioxidants with activity comparable to BHT and even higher [27].

Phenylpropanoids, to which anethole belongs, work either by direct scavenging of reactive oxygen species or by acting as chain-breaking peroxyl radical scavengers [28]. Anethole might chelate the zinc ion present in the catalytic site of MMPs and directly inhibit their activities, as well as exerting its antioxidant effects and inhibit their activity by attenuating oxidative stress. This is because oxidative stress activates nuclear factor Kappa B (NF-κB), an oxidant sensitive transcriptional factor, which plays a crucial role in the expression of MMP-2 as well as type I collagen [24, 29]. It has been determined that anethole at a concentration of 1 mM completely blocks NF-κB activation induced by TNF, phorbol ester, ceramide, or okadaic acid and partially by H_2_O_2_ [7]. In contrast, in our study, anethole was effective at a concentration lower than 1 µM. Anethole and its sulfated analogs have also been shown to increase the level of cellular glutathione (GSH) which, as an endogenous antioxidant, plays a key role in the protection against ROS damage [30].

It is worth mentioning that the majority of recently introduced antioxidants is hydrophilic, which inhibits membrane passage and their antioxidant applications. Essential oils can be absorbed through the skin. This process is not active but occurs by simple diffusion, therefore, volatile components must be in contact with the skin for a certain period of time. They also reach the bloodstream, and, for example, anethole is expelled with the air breathed out after about 20–40 min. Anethole is a small, lipophilic molecule which permeates through cell membranes. It is worth noticing that (*E*)-anethole is non-toxic, non-irritant, and non sensitizing. It has no genotoxic activity and is not significantly carcinogenic [31].

In conclusion, the results of the present study indicate that anethole exhibits protective properties against hydrogen peroxide-induced toxicity and collagen metabolism changes in human skin fibroblast cultures, which can suggest its therapeutic properties in oxidative stress-related skin diseases. However, further study is needed to elucidate the exact mechanism of this action.
